# Observational Studies: Getting Clear about Transparency

**DOI:** 10.1371/journal.pmed.1001711

**Published:** 2014-08-26

**Authors:** The PLOS Medicine Editors

## Abstract

The *PLOS Medicine* Editors endorse four measures to ensure transparency in the analysis and reporting of observational studies.

*Please see later in the article for the Editors' Summary*

## Extending the Standard

When publishing observational research, what information should journals make available to the medical community before a result can be considered sufficiently reliable to inform patient care or health policy?

For clinical trials, editors and researchers share the context of a reporting standard (CONSORT and its modifications [1]), a requirement for prospective, public registration of clinical trials [2], and a number of laws (e.g., [3]) and policies requiring data sharing. This combination supports transparency in the design, conduct, and reporting of clinical trials, and has proven sufficiently flexible to allow editors to define appropriate exceptions (e.g., [4]). Systematic reviews and meta-analyses, which often present a basis for clinical decisions, are also subject to strict reporting guidelines [5]. Public registration is available for such studies, and *PLOS Medicine* has long encouraged publication of the review protocol alongside the study report [6].

Should a similar framework apply to observational studies? In a Guidance and Guidelines article published recently in *PLOS Medicine*, Peat and colleagues [7] identify study registration, protocol publication, better study reporting, and data sharing as key to improving the transparency of prognosis research, which encompasses both observational and interventional studies. Case-control, cohort, and cross-sectional studies, even when given ethics review, are not subject to all of the regulations that facilitate standardized reporting of clinical trials. Nonetheless, when observational studies provide the best evidence available, they influence clinical practice. Selective presentation of analyses or non-publication of results can therefore misinform patient care. As the authors of the STROBE guidelines for reporting observational studies have noted, “Research should be reported transparently so that readers can follow what was planned, what was done, what was found, and what conclusions were drawn” [8].

## New Guidelines for Observational Studies in *PLOS Medicine*

The *PLOS Medicine* editors, in recent consultation with our editorial board, endorse measures in four areas to advance transparency in the analysis and reporting of observational studies.

### Quality of Study Reporting

*PLOS Medicine* already requires CONSORT checklists for the clinical trials that we consider, and our current author guidelines encourage authors to report observational studies according to the STROBE statement or its more specialized derivatives [8],[9]. Additionally, we endorse the following measure:

**(1) Going forward** ***PLOS Medicine*** **will require that the STROBE checklist for cohort, case-control or cross-sectional studies** [**10**] **, and the STARD checklist for studies of diagnostic accuracy** [**11**] **are included with manuscript submissions and published alongside reports of observational studies to which they apply. Authors must complete the appropriate reporting checklist not only with page references, but also with sufficient text excerpted from the manuscript to explain how they accomplished all applicable items.**

(Note: The EQUATOR Network (www.equator-network.org) provides access to reporting guidelines for additional specific study types, and authors within the particular research communities to which they apply are encouraged to use these guidelines when appropriate.)

### Data Sharing

The PLOS data policy [12] lays out requirements for data sharing in research manuscripts submitted after March 1, 2014. Therefore,

**(2) The PLOS Data Policy applies to all research submitted to** ***PLOS Medicine*** **, including observational studies.**

### Transparency Regarding What Was Planned and What Was Done

Research reports should make clear the distinction between hypothesis testing and exploratory evaluation of research questions, each of which may have validity, and both of which may occur in the same manuscript. Consequently,

**(3) Going forward,** ***PLOS Medicine*** **will require that reports of observational studies clearly specify the following items:**

**(a) What specific hypotheses the researchers intended to test, and the analytical methods by which they planned to test them;**
**(b) What analyses they actually performed; and**
**(c) When reported analyses differ from those that were planned, authors must provide transparent explanations for differences that affect the reliability of the study's results.**

For example, if a reported analysis was performed based on an interesting but unanticipated pattern in the data, authors must be clear that the analysis was data-driven. If hypotheses that were not included in the original study design later became important to test because new evidence became available from other studies, the authors should explain the situation, so that reviewers and readers need not wonder if the additional analyses were driven post-hoc by the authors' own data.

### Protocol Publication

*PLOS Medicine* already requires that study protocols be submitted with reports of clinical trials, and publishes these alongside the accepted paper. Accordingly, for observational studies,

**(4) Going forward, if a prospective analysis plan (from the study's funding proposal, IRB or other ethics committee submission, study protocol, or other planning document written before analyzing the data) was used in designing an observational study, authors must include the relevant prospectively written document with the manuscript submission for access by editors and reviewers and eventual publication alongside the accepted paper.**

As with studies of any design, in some cases the final analysis of an observational study will necessarily differ from the analysis plan (as a result of unforeseen practical circumstances, changes requested by peer reviewers, etc.). Under such conditions, authors should explain why analyses could not be completed as planned, or why they had to be revised, and thereby address potential concerns over selective non-publication. If no prospectively written document exists, authors should explain how and when they determined the analyses being reported.

## Looking Forward: Registration?

Editors cannot anticipate, nor would we desire to create rules for, every possible situation. Ultimately readers must judge the reliability of a study relative to their own needs and criteria.

We believe that promoting transparency by publishing prospective analysis plans and accounting clearly for substantial changes will help readers (including editors and peer reviewers) to assess the reliability of published observational research. Our goal is not only to establish these practices for *PLOS Medicine* but to advocate for such transparency as a standard within the research community.

We also recognize that no practice that applies only to published reports can guard against bias due to selective non-publication of observational studies, an issue that the open question of prospective protocol registration could potentially address [13]–[16]. ClinicalTrials.gov, among other registries, already allows registration of observational studies, and, as technologies allowing the “flexibility to update date-stamped protocols” [8] become more widely available, it seems likely that community standards for registration will move toward the greater transparency that such tools facilitate. At the same time, new evidence published by Dwan and colleagues in a recent issue of *PLOS Medicine* indicate that, in the case of clinical trials, substantial discrepancies exist between registered and reported analyses [17]. This result suggests that prospective registration alone, should it become the standard for observational clinical research, will still be no substitute for a conscientious explanation of differences between registered and actual analyses.

Peat and colleagues have contributed to this debate by calling for public registration of prognostic research, arguing that “routine registration of all prognostic studies, linked to an accessible study protocol using agreed reporting guidelines, would improve transparency and promote data sharing” [7]. They further point out that protocol sharing and registration are not equivalent: “Protocols describe the rationale, objectives, design, methodology, statistical considerations, and organization of a study and they present a research plan made before the conduct of the study. Compared to study registers, protocols contain more detail, particularly about study design and analysis plans.” Interestingly, while registration of clinical trials is mandatory under International Committee of Medical Journal Editors (ICMJE) requirements, posting of trial protocols in registries is optional [2].

The *PLOS Medicine* editors welcome opportunities to collaborate with the research community on issues of transparency and publication quality. We hope authors will find our requirements constructive and will let us know of suggested improvements. **We encourage members of the research community to share your insights by commenting on this editorial, either on the** ***PLOS Medicine*** **site or in Pub Med Commons, with thoughts on whether or how medical journals should establish requirements for prospective, public registration of observational studies that are intended to inform patient care or health policy.** We plan to revisit this issue in due course, and your views will help us determine our future direction.
